# Association of frailty with endothelial dysfunction and its possible impact on negative outcomes in Brazilian predialysis patients with chronic kidney disease

**DOI:** 10.1186/s12882-015-0150-1

**Published:** 2015-09-23

**Authors:** Henrique Novais Mansur, Júlio César Moraes Lovisi, Fernando Antonio Basile Colugnati, Nadia Rezende Barbosa Raposo, Natália Maria da Silva Fernandes, Marcus Gomes Bastos

**Affiliations:** IMEPEN Foundation, Federal University of Juiz de Fora, Juiz de Fora, Brazil; Academic Centre of Vitoria, Federal University of Pernambuco, Vitória de Santo Antão, Pernambuco Brazil; Federal University of Juiz de Fora, José Lourenço Kelmer Street, 1300, Juiz de Fora, Minas Gerais Brazil

**Keywords:** Chronic kidney disease, Endothelium, Frailty, Mortality

## Abstract

**Background:**

Frailty is a state of physiological vulnerability common in the elderly. It is more predominant in patients with Chronic Kidney Disease in comparison to healthy subjects, which can also be diagnosed in non-elderly individuals and be associated with innumerous causes such as muscle strength, body composition and inflammation. The association between frailty and endothelial function, as well as the association between frailty and the combined outcome of mortality multiple cause and start of renal replace therapy were assessed.

**Methods:**

In the initial analysis, sixty-one predialysis patients with Chronic Kidney Disease stages were evaluated and included in this study. Due to patient drop-out during follow-up, fifty-seven patients were subsequently re-evaluated 12 months later. The diagnosis of frailty was based on the Johansen et al. (J Am Soc Nephrol 18(11):2960-67, 2007) criteria. The groups were divided into Non-frail and Frail. Sociodemographic, inflammatory markers (IL-6, TNF-?, CRP-us), endothelial dysfunction (flow-mediated vasodilatation - FMD), body composition (DXA) and the 25-hidroxi-vitamin D parameters were analyzed.

**Results:**

The average age of the patients used in the study was 64.9 ± 10.3 years old. The predominance of frailty was 42.6 %, of which 46 % were non-elderly. After some adjustments, frailty was associated with gender (OR = 11.32; IC 95 % = 2.30 to 55.67), advanced age (OR = 4.07; IC 95 % = 1.02 to 16.20), obesity (OR = 6.63; IC 95 % = 0.82 to 11.44) and endothelial dysfunction (OR = 3.86; IC 95 % = 1.00 to 14.88). The ratio of the incidence of frail subjects to the variable outcome was 2.5 (CI 95 %, 1.04 to 6.50).

**Conclusions:**

Although an observational study does not allow one to determine the casual relation between frailty and endothelial dysfunction, we conclude that frailty was predominant in our sample of Brazilian patients with chronic kidney disease on predialysis, even in elderly individuals. This was linked to either worse endothelial function or mortality.

## Background

Frailty is a multidimensional syndrome that features a loss of physiological reserves and is commonly related to advanced age, conferring a state of vulnerability among elderly people [1]. Frailty is manifested through a loss of both weight and strength, as well as exhaustion and low capacity to exercise, and thereby places the afflicted individual at increased risk of falls, hospitalization and death [2].

Patients with chronic kidney disease (CKD) are more susceptible to frailty, probably owing to anemia, inflammation, dyslipidemia, and osteoarticular alterations such as bone and mineral disorders and muscle dysfunction. In addition, CKD patients frequently present with cardiovascular comorbidities, mainly secondary to endothelial dysfunction, which clinically present as left ventricular hypertrophy and reduced coronary perfusion pressure [3].

Frailty has been studied in patients with CKD, mainly those undergoing dialysis [4, 5], with a few publications concerning predialysis patients [4, 6, 7]. In the only prior study carried out in Brazil, we showed that frailty is prevalent among predialysis patients, and is found even among non-elderly individuals [8]. However, the relationship between frailty and endothelial dysfunction remains unknown, even among individuals with CKD.

The goals of the present study are to evaluate the relationship between frailty and endothelial dysfunction in Brazilian predialysis patients with CKD, and to evaluate the impact of frailty on all-cause mortality and the need for renal replacement therapy (RRT).

## Methods

### Study population

This study was approved by the Ethics Committee on Research of the Federal University of Juiz de Fora and informed consent was obtained from all patients prior to enrollment in the study. The procedures were in accordance with the ethical standards of the responsible committee on human experimentation and with the Helsinki Declaration.

The patients, sampled by convenience, were recruited from the CKD outpatient clinic of the IMEPEN Foudation of the Federal University of Juiz de Fora, Brazil. All patients were diagnosed with CKD stages 3–5 and were on predialysis. The diagnosis and categorization of CKD was established according to the new CKD guidelines [9]. The glomerular filtration rate (GFR) was estimated from the serum creatinine level using the equation proposed by the Modification of Diet in Renal Disease Study [10].

Individuals were excluded from the study if they presented with severe neuropathy, gout, amputation, severe physical sequelae caused by stroke, deep vascular thrombosis, Parkinson’s, pregnancy, chronic pulmonary obstruction disease, neoplasia, human immunodeficiency virus infection, or cognitive impairment as determined by the Mini Exam Mental State [11].

**Fig. 1:**
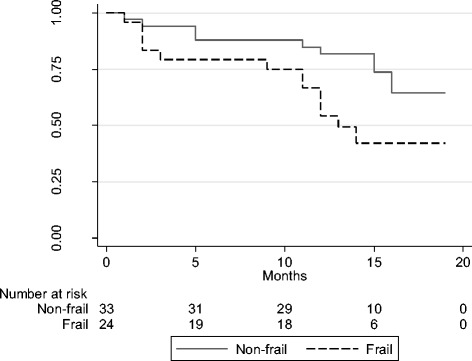
Fluxogram of the sampling selection process used in the study

Between June 2011 and September 2012, 380 patients were seen in the CKD clinic and 97 were considered eligible for this study. However, 17 of these patients did not agree to participate in the research, 9 had already started dialysis, 8 died, and 2 could not be located, amounting to a total of 61 patients who were included in the study. At the 12-month follow-up, 1 patient withdrew his consent, another patient moved to another city, and 2 patients could not be located, totaling to 57 patients being included for the follow-up analysis (Fig. 1).

### Assessment of frailty

To evaluate frailty, we used the criteria proposed by Johansen et al. [5] It is a criteria similar or identical to the modification by Woods et al. [12] of Fried’s criteria for frailty^2^.

Scores were assigned for each criterion as follows: muscle weakness (a score of 75 points on the Physical Function scale of the Medical Outcomes Study 36 – Item Short, 2 points; exhaustion (a score of 55 on the Vitality scale of the SF-36), physical inactivity (patients who reported that they “almost never or never” exercised were classified as inactive), and unintentional weight loss (ascertained using information available in the medical chart within 30 day before study entry), 1 point each; this could total to a maximum of 5 points. Frailty was diagnosed when patients were assigned ≥3 points.

We have chosen the Johansen’s criteria because it has already been used in CKD patients and it uses direct questions and issues presented in the SF-36, a questionnaire validated in the Brazilian population.

### Measurement of other variables

Data on the etiology of CKD and the medications prescribed were obtained from the medical records. The body mass index (BMI) was assessed by dividing the weight in kilograms by the height in meters squared. Body composition and bone health were assessed using dual-energy X-ray absorptiometry (DXA) of the whole body, and the data generated were analyzed using LUNAR EXPERT software, version 8.2 (GE LUNAR). Abdominal circumference was measured as proposed in the Third Report of the Expert Panel on Detection, Evaluation, and Treatment of High Blood Cholesterol in Adults (Adult Treatment Panel III) [13].

Laboratory tests were performed on blood samples collected after 12 h of fasting. Part of the sample was stored at −80 °C for assessment of tumor necrosis factor-α (TNF-α; DuoSet R&D Systems, Minneapolis, MN, EUA) and interleukin-6 (R&D Systems, Minneapolis, MN, USA) levels following the protocols suggested by the suppliers.

The 25-hydroxyvitamin D (25-OH-D) level in the serum was determined using a high-performance liquid chromatography method [14], and was considered adequate at levels above 30 ng/mL.

### Endothelial function

Endothelial function was assessed by an observer blinded to the patient’s clinical status, using a Philips Envisor ultrasound system, equipped with a high-frequency probe (7–12 MHz.) and specific software for cardiac and vascular analysis. Fasting individuals were examined in the morning; caffeine consumption and smoking were not recommended on the day of the exams. Among female patients, no evaluation was performed during the menstrual cycle to avoid possible estrogen influence on endothelial behavior.

The patients were requested to maintain an idle position for at least 10 min in a temperature-controlled room. Electrocardiographic monitoring was carried out on channel 1 (CM5). The brachial artery was insonated in a longitudinal plane in the antecubital fossa. The cuff of a blood pressure monitor was placed on the arm. Anatomical markers were used for proper positioning of the transducer in order to obtain electrocardiographic measurements from the brachial artery using R wave amplitudes. Doppler ultrasound was also performed. After assessing the basal images, bi-dimensional and Doppler, the cuff was inflated to suprasystolic pressure (50 mmHg above systolic pressure) for 5 min. The cuff was then released quickly and either the bi-dimensional images or Doppler evaluation of the brachial artery was recorded for 5 min.

Flow-mediated vasodilatation (FMD) was assessed using the maximum diameter of the artery obtained as a percentage of the baseline. Reactive hyperemia was calculated using the flow change as a percentage of the baseline.

Values of less than 10 % of the variability found among 5 measures of pre- and post-pressure hyperemia were adopted as the cutoff for normal FMD.

### Follow-up

**Fig. 2:**
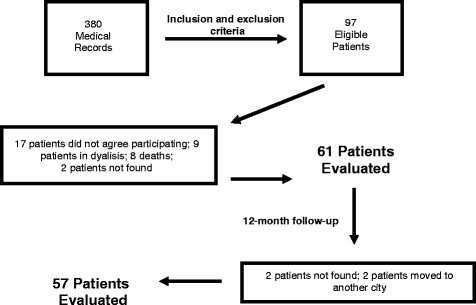
Kaplan–Meier survival estimates between frail and non-frail subjects

After 12 months, the patients or their relatives were contacted by telephone and were questioned about hospitalization history, need for RRT, and death. Need for RRT or death was classified as the combined variable outcome. Patients who survived and were not on dialysis or did not receive any transplants were re-characterized for frailty (Fig. 2).

### Statistical analysis

For statistical analysis, STATA 11.0 ( STATACorp LP. College Station, Texas) software was used. Descriptive statistics for the mean with the standard deviation or the median with the interquartile interval were applied as necessary.

After using the Kolmogorov-Smirnov normality test, the differences between the frail and non-frail groups were evaluated. The student’s *t*-test was used for independent groups when the normality test was not rejected; otherwise, the Mann–Whitney *U* post-hoc test was used. The chi-squared test was used for analysis of categorical variables.

The correlation between the obtained scores of frailty, which used an ordinal scale of 6 points (0–5 points), and the variables of the study was assessed using the Spearman correlation. The variable frailty, which is dichotomous, was further analyzed by simple/bivariate logistic regression models. For the multivariate model, all the explicative variables that presented a chance of statistical significance less than 20 % were excluded, but those presenting significant statistical differences (*p* < 0,05) were included in the model.

Survival analysis was used to assess outcomes and it included the number of months reported by the individuals since the first appointment. In case of a lack of outcome, 12 months was used as a temporary horizon, and the observed data was censured. To verify the difference among the distribution of survival function, the Kaplan–Meier approach with log-rank tests was used. The hazard ratio was estimated by Cox regression models adjusted according to gender and age.

## Results

### Characteristics of the cohort

At baseline, the average age of the patients was 60 ± 11.5 years; 41 % were women, 54.1 % were non-Caucasian, 27.9 % obese, and 14.8 % smokers. Hypertension was found to be the main cause of CKD (29 %) and the main associated comorbidity (57.4 %). The average serum creatinine level was 2.3 (1.7–3.5) mg/dL and the average estimated GFR was 23 (16.0–39.0) mL/min/1,73 m^2^ (Table 1).

**Table 1:** Descriptive analysis of demographic, laboratorial and clinical data

	Total (*n* = 61)	Non-frail (*n* = 35)	Frail (*n* = 26)	*P* value
Age (years)	60.5 ± 11.5	57.3 ± 11.4	64.9 ± 10.3	0.009*
Female - n (%)	25 (41.0 %)	10 (28.6 %)	15 (57.7 %)	0.02*
Non-Caucasian - n (%)	33 (54.1 %)	22 (62.9 %)	11 (42.3 %)	0.11
Smoking - n (%)	9 (14.8 %)	5 (14.3 %)	4 (15.4 %)	0.90
BMI classification - n (%)				0.362
Malnourished ( <18,4 kg/m^2^)	2 (3.3 %)	1 (2.9 %)	1 (3.8 %)	
Eutrophic (18,5 a 24,9 kg/m^2^)	20 (32.8 %)	14 (40 %)	6 (23.1 %)	
Overweight (25,0 a 29,9 kg/m^2^)	22 (36.1 %)	13 (37.1 %)	9 (34.6 %)	
Obese ( ≥30,0 kg/m^2^)	17 (27.9 %)	7 (20 %)	10 (38.5 %)	
Fat mass (kg) – mean (SD)	22.1 ± 8.2	20.3 ± 7.4	24.6 ± 8.7	0.05*
Fat-free mass (kg) – mean (SD)	46.6 ± 11.3	48.5 ± 9.7	44.1 ± 12.9	0.148
Abdominal circumference – mean (SD)	94.14 ± 13.6	91.6 ± 13.0	97.4 ± 13.9	0.107
Comorbidities – n (%)				0.235
Hypertension	35 (57.4 %)	17 (48.6 %)	18 (69.2 %)	
Diabetes Mellitus	1 (1.6 %)	1 (2.9 %)	0 (0 %)	
Hypertension and Diabetes Mellitus	11 (18 %)	6 (17.1 %)	5 (19.2 %)	
Not present	14 (23 %)	11 (31.4 %)	3 (11.5 %)	
Osteopenia/Osteoporosis – n (%)				0.01*
Osteopenia	12 (19.7 %)	8 (22.9 %)	4 (15.4 %)	
Osteoporosis	6 (9.8 %)	0 (0 %)	6 (23.1 %)	
Creatinine (mg/dL) ^a^	2.3 (1.7–3.5)	2.2 (1.6–3.4)	2.4 (1.8–3.6)	0.53
GFR (mL/min/1,73 m^2^) ^a^	23 (16.0–39.0)	28 (18.0–41.0)	22 (14.7–32.2)	0.15
Glicemia (mg/dL) ^a^	91 (83.5–102.5)	92 (86.0–100.0)	88.5 (79.5–104.2)	0.79
iPTH (pg/ml) ^a^	149.3 (95.4–358.5)	127.9 (84.1–264.1)	248.8 (124.3–409.7)	0.02*
TSH (μUI/mL) ^a^	2.2 (1.4–3.1)	2.4 (1.4–3.3)	2.0 (1.3–2.7)	0.70
Total cholesterol (mg/dL) – mean (SD)	178.0 ± 38.4	177.1 ± 36.9	179.3 ± 41.0	0.83
Ferritin (ng/dL) ^a^	126.4 (79.1–202.6)	103.6 (71.3–209.9)	128.4 (89.1–202.2)	0.47
TSAT (%) – mean (SD)	34.1 ± 12.5	37.1 ± 12.9	30.1 ± 11.1	0.02*
Hemoglobin (g/dL) – mean (SD)	12.9 ± 1.7	13.3 ± 1.8	12.4 ± 1.6	0.07
TNF-α (pg/ml) ^a^	9.7 (5.6–29.5)	10.0 (6.5–35.8)	8.6 (5.5–16.2)	0.27
IL-6 (pg/ml) ^a^	2.7 (1.9–4.5)	2.3 (1.8–4.1)	3.2 (1.9–4.6)	0.22
CRP (mg/L) ^a^	2.1 (1.3–4.1)	1.7 (1.1–4.2)	2.4 (1.5–4.1)	0.28
Vitamin D (nmol/L) – mean (SD)	21.9 ± 3.8	22.1 ± 3.6	21.5 ± 4.1	0.52
Calcium (mg/dL) – mean (SD)	9.8 ± 1.1	9.8 ± 0.9	9.8 ± 1.4	0.79
Phosphorus (mg/dL) – mean (SD)	3.8 ± 0.9	3.8 ± 0.8	3.8 ± 1.0	0.95
Calcium x phosphorus product (mg/dL) – mean (SD)	37.2 ± 9.0	36.4 ± 8.7	38.2 ± 9.4	0.45

^a^Non-normal variable: data presented as median (Interquartil Interval)

- Normal variable: mean ± standard deviation

^*^ *p* < 0,05

*FMD* flow-mediated vasodilatation, *ESA* eritropoiesis-stimulating agent, *GFR* glomerular filtration rate, *iPTH* Intact parathyroid hormone, *TSH* Thyroid-stimulating hormone, *TSAT* Transferrin saturation index, *TNF-α* Tumor necrosis factor-alfa, *IL-6* Interleucin 6, *CRP* C-reactive protein (ultrasensitive) test

### Prevalence and characteristics of frail patients

The prevalence of frailty in the study group was 42.6 and 46 % of these cases occurred among non-elderly patients. Compared to the non-frail group, the frail group mainly comprised women and patients of a higher average age. The frail group also comprised a higher number of Caucasian subjects, although there was no statistically significant difference when the 2 groups were compared. The prevalence of tobacco use too did not differ between the groups.

There was no difference between the estimated GFR and number of patients per category of CKD when the frail and non-frail groups were compared.

The analysis of body composition showed that the BMI was similar in both groups, that there was no difference in the abdominal circumference between the groups, and that frail patients presented with an increased amount of fat mass, but not fat-free mass. Further, the incidence of osteoporosis in the frail group was 100 %, compared to the 15.3 % in the non-frail group.

Hypertension and diabetes were the main causes of CKD in frail patients. The occurrence of hypertension (69.2 %), although high, was not different between the 2 groups. We observed higher values for serum intact parathyroid hormone (iPTH) and transferrin saturation index (TSAT) in the frail patients, but no significant difference was observed for thyroid-stimulating hormone, total cholesterol, ferritin, hemoglobin, 25(OH)D, calcium, phosphorus, bicarbonate, albumin, ultra-sensitive C-reactive protein (CRP-us), interleukin-6 (IL-6), or TNF-α between the groups.

### Correlation between frailty and laboratory and clinical data

Among the laboratory and clinical measurements, only fat mass (*r* = 0.25; *p* = 0.05), endothelial function (*r* = −0.367; *p* = 0.004), iPTH (*r* = 0.30; *p* = 0.01) TSAT (*r* = 0.1475; *p* = 0.257) and hemoglobin-b (*r* = −0.092; *p* = 0.479) were not correlated with frailty.

### Association between frailty, FMD and others variables

The frail group had 9 subjects with FMD ≥ 10 % (34.6 %) and the non frail had 21 subjects with FMD ≥ 10 % (60 %).

In the bivariate analysis, the variables found to be associated with frailty were: female gender (odds ratio [OR] = 3.41; 95 % confidence interval [CI] = 1.17–9.93), age over 60 years (OR = 3.00; 95 % CI = 1.03–8.73), and endothelial dysfunction (OR = 2.83; 95 % CI = 0.99–8.13) (Table 2).

**Table 2:** Analysis of raw odds ratio

Variables	OR	*P* value	IC 95 %	
Female	3.41	0.03	1.17	9.93
>60 years	3.00	0.04	1.03	8.73
Overweight (BMI: 25 – 29.9 kg/m^2^)	1.48	0.53	0.43	5.10
Obese (BMI ≥ 30 kg/m^2^)	3.06	0.10	0.82	11.44
Abdominal circunference (H ≥ 94 cm; M ≥ 80 cm)	2.48	0.14	0.75	8.18
Anemia (Hb ≤ 11 g/dL)	1.39	0.71	0.25	7.52
Glycemia (100 mg/dL)	1.81	0.29	0.60	5.40
Phosphorus^a^	0.24	0.21	0.02	2.19
Calcium (8,4 a 9,5 mg/dL)	0.77	0.63	0.28	2.17
iPTH^b^	1.62	0.47	0.43	6.14
TSH (4,0 μUI/mL)	2.00	0.43	0.36	11.23
Serum albumin (3,5 mg/dL)	2.00	0.19	0.70	5.69
Total cholesterol (200 mg/dL)	1.28	0.66	0.42	3.96
LDL-c (130 mg/dL)	1.09	0.91	0.26	4.54
HDL-c (H ≥ 60 mg/dL; M ≥ 50 mg/dL)	0.64	0.42	0.22	1.88
TGL (150 g/dL)	0.79	0.67	0.28	2.28
TSAT (≥20 %)	0.51	0.41	0.10	2.53
Ferritin (≤100 ng/dL)	0.62	0.38	0.22	1.79
HCO3 (22 mmol/L)	1.02	0.97	0.37	2.85
FMD (dif ≥10 %)	2.83	0.05	0.99	8.13
TNF-α (pg/ml) ^c^	1.16	0.79	0.39	3.39
IL-6 (pg/ml) ^d^	1.25	0.49	0.66	2.35

^a^P (Stages 3 and 4: 2.7 to 4.6 mg/dL, Stage 5: 3.5 to 5.5 mg/dL)

^b^iPTH (Stage 3: 35 a 70 pg/ml, Stage 4: 70 a 110 pg/ml, Stage 5: 110 to 300 pg/ml)

^c^TNF-α (<6.83 pg/ml; 6.83–19.22 pg/ml; >19.22 pg/ml)

^d^IL-6 (<2.13 pg/ml; 2.13–3.9 pg/ml; >3.9 pg/ml)

*FMD* flow-mediated vasodilatation

After adjusting for confounding variables, frailty was found to be associated with gender (OR = 11.32; 95 % CI = 2.30–55.67), advanced age (OR = 4.07; 95 % CI = 1.02–16.20), and obesity (OR = 6.63; 95 % CI = 0.82–11.44) (Table 3). The values for CRP-us and 25-OH-D are not described here because those patients with inadequate levels were all included in the frail group. Frailty was also associated with endothelial dysfunction (OR = 3.86; 95 % CI = 1.00–14.88).

**Table 3:** Multivariated regression analysis

Variable	OR	*P* > z	IC 95 %	
Female	11.32	0.00	2.30	55.67
Age > 60 years	4.07	0,05	1.02	16.20
Eutrophic	1	-----	-----	-----
Overweight	3.05	0.17	0.63	14.68
Obese	6.63	0.03	1.19	36.77
FMD	3.86	0.05	1.00	14.88

*FMD* flow-mediated vasodilatation

Variable used in multivariated regression: Anemia, waist circumference, glycemia, TSH, IL-6, PCR

### Association between frailty and negative outcomes

Re-evaluation of the patients at the 12-month follow-up, after adjusting for age and gender, revealed that frailty accounted for a hazard ratio of 2.5 (95 % CI = 1.04–6.10), a greater risk of negative health outcomes.

When evaluating the survival curves comparing the groups generated by the combination of frailty and FMD, the log rank test gives a *p*-value of 0.09. Although insufficient to reject the null hypothesis of homogeneity of the survival curves between the groups, draws attention that under the assumption of the same null hypothesis, the expected negative outcomes in the stratum formed by frailty and FMD <10 % value would be 5 events (provided by the estimates used in the log-rank test). However, we observe 10 events, twice the expected, what make us suppose about an interactive detrimental effect of frailty and FMD to explain the increased risks of outcomes observed, which did not reach a *p*-value <0.05 probably due to the small sample size.

## Discussion

Using a study cohort comprising Brazilian predialysis patients, we observed that frailty occurs at a high frequency; is prevalent even among non-elderly patients; is associated with gender, advanced age, obesity, and endothelial dysfunction; and that it identifies individuals with a higher chance of unfavorable outcomes, including need for RRT, hospitalization, or death.

Barreto [15] argued for the universality of evaluating frailty in any culture or country. The author justifies his conclusion on the basis of the fact that the causes of frailty are linked to biological deficits, most of which are due to advanced age, and therefore reflects the quantification of health vulnerability. On the other hand, such vulnerability may vary depending on genetic, behavioral, or social characteristics, supporting the idea of having a specific evaluation to assess frailty according to countries or regions. Moreover, even though frailty usually assesses such criteria, it also follows social, environmental, and psychological trends, emphasizing the necessity of evaluating frailty using customized instruments for a specific culture or local areas.

The comparison of studies on the prevalence of frailty is hindered by the innumerous diagnostic models of frailty as well as the different locations where the evaluations took place. Santos-Eggimann et al. [16] compared frailty prevalence in 10 European countries and found prevalences that varied from 3.9 to 21 %. In another multicenter study involving 5 countries in South America, Alvarado et al. [17] found prevalences of frailty that varied from 27 to 40 % in both genders. In Alvarado’s study, although the instrument employed was different from the instrument that we used, the authors described a prevalence of 40.6 % among Brazilian participants, similar to that identified among our patients (42.6 %).

Irrespective of the instrument chosen for assessing frailty, the majority of studies linking frailty to CKD shows an association between frailty, advanced age [4, 5], and female gender [4]. Our data show that 46 % of the frail population was less than 60 years old, a finding in agreement with the data obtained in another study performed by our group, which included patients undergoing dialysis. These findings are similar to those described in American patients on dialysis, where the same instrument was used for assessment of frailty [5].

More recently, Roshanravan et al. [7] used the criteria of frailty described by Fried et al. [2] and found comparable results in predialysis patients in the USA. Taken together, these data point towards a possible association of frailty with inflammation and endothelial dysfunction, both also common in patients with CKD.

Although weight loss is one of the components of frailty, it was found in only 4 (11.5 %) of our patients; the frail group presented an average serum albumin level of 3.5 ± 0.3 g/dL. Multivariate analysis identified a strong association between obesity (assessed by DXA) and frailty. Together, these findings suggest that frailty, in this group of patients, was not due to undernourishment.

Excess weight can be associated with some of the criteria used and with the risk factors typically associated with frailty. Obese patients demonstrate impaired physical function and become exhausted more easily [18, 19]. Additionally, obesity can prompt a pro-inflammatory condition, common in frail individuals [20, 21]. The direct relationship between frailty and obesity was found by Fried et al. [21] in frail older women, who presented more subcutaneous fat, as assessed through skin fold tests. Similarly, Sanders et al. [22] have also reported an association between excess weight, assessed through BMI, and frailty, assessed by a 10-point scale of frailty. On the other hand, Alvarado et al. [17] did not find any association between high BMI and frailty in a Latin–American elderly population. We also did not find any association between frailty and BMI, although compared to the non-frail group, the frail group comprised less eutrophic (23.1 × 40 %) and more obese (38.5 × 20 %) patients.

Nevertheless, Hubbard et al. [23] noticed a relationship between BMI and frailty when the subject was undernourished or overweight and obese, especially in those presenting larger abdominal circumference. In our study, the abdominal circumference was not statistically different among the groups, but it is likely that there is a clinical difference among them (frail = 97.4 ± 13.9 cm; non-frail = 91.6 ± 13.0 cm).

In our study, there was no statistical difference between frail and non-frail patients regarding muscle mass, possibly due to the absence of metabolic acidosis and a good nutritional state.

Notably, osteoporosis was documented (23 %) only in patients with frailty, since we have found no prior studies associating frailty with bone mineral disorders in patients with CKD.

One of the factors that can explain such relationships is the secondary hyperthyroidism that was more frequently observed in the frail group, which was also correlated to the criteria of frailty, although it lost statistical strength following adjustments. The other variables associated with bone and mineral metabolism (calcium, phosphorus, and vitamin D), and were different between the groups, although not significant, were shown to be protective factors according to the bivariate analysis. It is also worth noting that glucocorticoid and vitamin D use was not different between the groups, and they probably did not influence the relationship between frailty and bone metabolism.

Vitamin D has been associated with frailty in many studies on elderly populations [24–26]. Nonetheless, in our study, there was no association between frailty and vitamin D, probably due to the fact that both groups presented vitamin D insufficiency, even though the patients resided in a sunny country. Our findings are in accordance with studies reported by Cuppari et al. [27] and Diniz et al. [28], who also observed lower levels of vitamin D in the Brazilian population with CKD.

Perhaps the most relevant result identified here was the strong association of endothelial dysfunction with frailty that, to the best of our knowledge, has not been reported in the literature.

The association between endothelial function and frailty might be due to the sympathetic hyper-activation of CKD [29], generating an autonomous dysfunction and, consequently, a reduction of vascular perfusion in the skeletal muscle. However, the evaluation of sympathetic activity was not an objective of the current study.

Another hypothesis for the association between frailty and endothelial dysfunction is oxidative stress, which is related to endothelial dysfunction in patients with CKD [30]. According to an animal study, the increase in oxidative stress, coupled with the reduction in nitric oxide production, may generate a reduction in nitric oxide bioavailability, thereby inducing endothelial dysfunction [31].

In our study, only 34.6 % of the patients presented with FMD greater than 10 % of the difference between the evaluation of pre- and post-hyperemia, while in the non-frail group, 60 % of the patients presented such values. When correlating endothelial function with the scores of frailty criteria, we found a strong negative association. Moreover, the risk of being frail when the FMD was less than 10 % was 2.83 according to bivariate regression analysis and 3.86 according to multivariate regression analysis.

In contrast to the majority of the studies that assessed the relationship between frailty and inflammatory markers, our results obtained using CRP-us, IL-6, and TNF-α did not demonstrate any such relationship, which could be due to the great variability observed in the results of these markers, reinforcing the existence of another association between endothelial function and frailty.

Because we have identified a strong association between endothelial function and frailty, and because we did not identify the same trend for inflammatory markers, we suggest another hypothesis that can explain the relationship between frailty and endothelial function: vascular calcification of the tunica media, common in patients with CKD [32], which is likely to produce a reduction in vascular perfusion. Despite the normal values of calcium and phosphorus reported in our study population, this hypothesis can be justified by the presence of hyperparathyroidism or even by the presence of free radicals, which is common to the referred population, although it was not assessed in our study.

The relationship between physical capacity and negative health outcomes in CKD remains unknown [33]. In terms of frailty, its relationship with negative outcomes is positive and well documented in the literature [2, 34–37]. However, few studies demonstrate the relationship between frailty and negative health outcomes in the CKD population [5–7, 38]. Among them, only 2 were conducted on non-dialysis patients [6, 7], but using only 1 creatinine estimation [7].

All the results describing frailty in patients with CKD, apart from the criteria of frailty used, acknowledge our results. Our data show that the negative outcomes were more common in frail patients (HR = 2.51; 95 % CI = 1.04–6.10). Wilhelm-Leen et al. [6] reported an HR of 2.21 (95 % CI = 1.49–3.28) for patients in stages 1 and 2 between frailty and mortality, 2.48 for patients in stage 3A (95 % CI = 1.57–3.93), and 5.58 for patients between stages 3B and 5 (95 % CI = 3.40–10.16). Bao et al. [39] and Johansen et al. [5] studied the same relationship between mortality and frailty in dialysis patients and found an HR of 1.75 (95 % CI = 1.44–2.24) and 2.24 (95 % CI = 1.60–3.15), respectively.

However, our study has some limitations. This was a single-centered study, and hence, comprised a small study sample. Further, the observational nature of this study did not allow for the identification of a causal relationship. Despite the use of known criteria to assess frailty, we consider the evaluation of physical activity less useful in determining the fenotype of frailty, although a high index of sedentary behavior was found.

## Conclusion

In conclusion, we documented an elevated frailty prevalence in a sample of Brazilian predialysis patients. We found frailty to be associated with gender (female), age over 60 years-old), obesity, and endothelial dysfunction. In addition, we found a high percentage of non-elderly patients with frailty. Nevertheless, prospective and randomized studies are necessary to investigate the cause-and-effect relationship between frailty and endothelial function, perhaps by assessing oxidative stress.
